# Effect of Minor Amendments of Patient’s Position on the Accuracy of Linear Measurements Yielded from Cone Beam Computed Tomography

**Published:** 2013-03

**Authors:** Sh Shahidi, A Feiz

**Affiliations:** a Dept. Oral and Maxillofacial Radiology, Biomaterial Research Center, School of Dentistry, Shiraz University of Medical Sciences, Shiraz, Iran; b Dept. Oral and Maxillofacial Radiology, School of Dentistry, Ahwaz University of Medical Sciences, Ahwaz, Iran

**Keywords:** Cone Beam Computed Tomography, Linear Measurement, Position

## Abstract

**Statement of Problem: **Image distortion in intra and extra-oral radiographs are an unavoidable phenomenon. Patient's positional changes from the routine alignments is an important issue for this unwanted alteration, Therefore the accuracy of the dimensional measurements will be affected.

**Purpose:** Our purpose is to find out the effect of minor changes (possibly happening in the clinic) in the position of a human dry skull on the accuracy of the measurements acquired by Cone Beam Computed Tomography (CBCT).

**Materials and Method: **In this study, 3 locations on the skull were pointed with radio- opaque markers. Imaging process with Kodak 9000 CBCT was performed in standard and 10 analytically miss- oriented positions. Then 2 distances were measured between the centers of markers. Later, these measurements were compared with the standard position values.

**Results: **There was not any imperative difference in the measurements of the 10 altered positions yielded in this study with standard position values.

**Conclusion: **According to our results, apparently, the accuracy of linear measurements in CBCT images is endorsed by unintentional small changes in the patient’s position during the projections.

## Introduction

During recent decades, several methods were invented to assess the maxillofacial area which among them, intraoral and extraoral radiography and recently Cone Beam Computed Tomography (CBCT) are more pertinent [1-2]. Spiral CT is usually recognized as a precise implement for constructing measurements of bony structures; however, they have some limits. The most important one is the two-dimensional evaluation of a three-dimensional structure. Compared with CT scans, In CBCT radiological examinations, the area is scanned with a single cone-shaped beam aimed to a flat panel detector. The flat panel detector certifies a high spatial resolution imaging with isotropic voxels (square cuboid) apparently unaltered by minor changes in the inclination of the patient [16]. Of these imaging techniques, CBCT is capable of assessing facial structures in three dimensions. As well as three-dimensional evaluation, CBCT is capable of producing images with high accuracy and resolution [3-4]. 

Initially in 1982, CBCT was developed for angiography and eventually was practiced for maxillofacial imaging. This technology uses a divergent or cone- shaped source of ionizing radiation and a two- dimensional image receptor. This provides successive images in a full scan of the surrounding area [5-6]. A radiographic image is considered ideal when different measurements are possible with high accuracy. There are several reasons for the failure to reach this issue; The most important is the image distortion [5].

Image distortion refers to the changes in size or shape and is referred as image size and image shape distortion [5]. Two major factors are involved in the extra oral radiographic image distortion; the patient position and the device arrangements [7].

The mechanism of tube rotation in routine employment of CBCT devices resembles the panoramic machines and the rotation of the radiation unit against the image receptor causes the distortion originally.

The software of the device uses a special algorithm that expels errors resulted from the distortion just before the final image reconstruction, thus the resolution and the accuracy of the images do not alter [5, 8].

According to our knowledge only three studies reported the effects of pre-planned changes in the position of human dry skull with CBCT.

Ludlow et al., in a part of their survey, studied the relationship between the changes in the position of head and the linear measurements of images obtained from CBCT. Their study was conducted on dry human skull then two rotational and linear changes in position were surveyed. Results show the fact that no significant differences exist between linear measurements compared to the ideal position [9].

Hassan et al. compared the accuracy of linear measurements of three- dimensional images obtained from CBCT with two-dimensional images acquired from conventional radiographs and concluded that changing the position of the head in terms of  rotation,  does not affect the accuracy of linear measurements [10]. Berco et al., as a part of their study, evaluated the relationship between changes in the position of head on linear measurements obtained from CBCT images. Their study was performed on a human dry skull and determined that the changes in the position of the skull (only in a rotating position) compared to the ideal position had no significant effect on linear measurements [11].

The aim of this research is a comprehensive survey of pre-planned changes in the position of human dry skull, and their effect on the accuracy of linear measurements in the images obtained from CBCT.

## Materials and Method

In this study, human dry skull was used for imaging.

**Preparation of skull**

**Figure 1:**
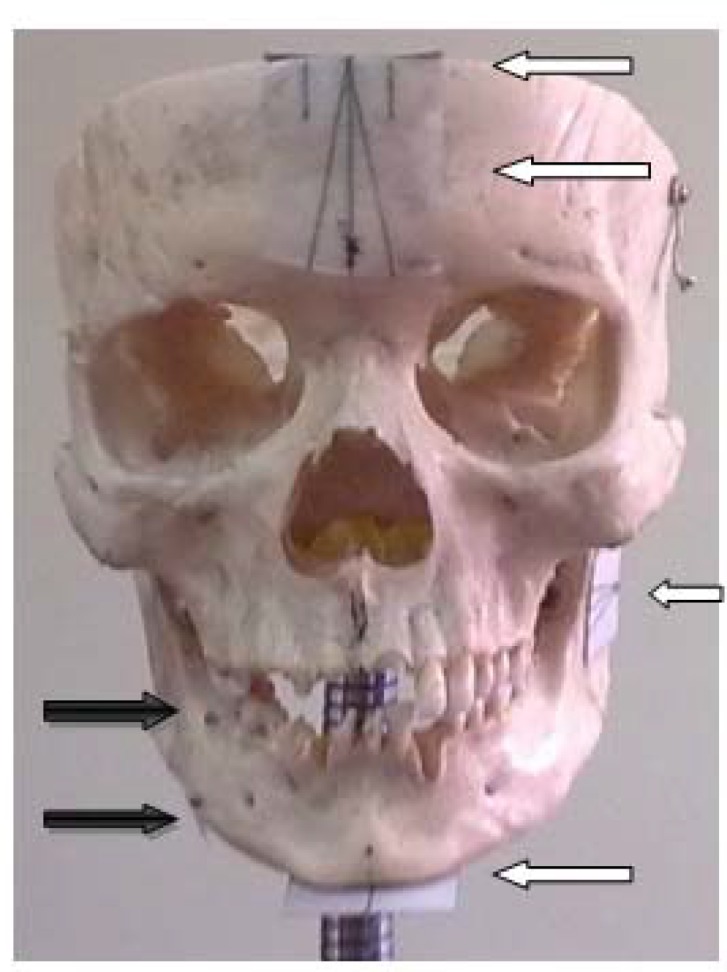
The black arrow position of the markers. White arrows location of the pages

Three Radio-opaque spherical markers, made ​​of lead (with a diameter of 5.2 mm) (Figure 1), were placed in three different locations, as follows:

- Marker 1: on the buccal area between first and second right mandibular molars at the level of alveolar crest.
- Marker 2: at the level of marker1, the same region the lingual side.
- Marker 3: In line with marker 1 on the buccal side of inferior border of the mandible.

Four small sheets of paper were installed on the skull, containing linear measurements and angles required for positioning (Figure 1).

The locations of these sheets are as follows:

1. On the frontal area of the skull, parallel with the axial plane. Midsagittal line and two other lines with 10° angle to the first line drawn on it. (The right line was named "a" and the left line "b"). Also a line (c) perpendicular to the first line was drawn with a distance of 7 mm from the frontal surface of the skull.
2. On the frontal area of the skull, parallel with the coronal plane which midsagittal line and two other lines with 10° angle to the midsagittal line were drawn on it. (The right line was named "d" and the left line "e"). Also, two parallel lines with midsagittal and each line with a distance of 7mm were drawn

(The right line was named "f" and the left line "g").

1. On the mandibular ramus parallel to the sagittal plane, a line parallel to the mandibular occlusal plane and two lines with 10 degree angle to the first line was drawn. (The upper line was named" and the lower line "I").
2. Below the chin and parallel to the axial plane, a line, along the midsagittal plane was drawn. One line (j) perpendicular to the aforementioned line parallel to the external surface of the chin was traced with a distance of 7mm.

The skull was then installed in the holder with the ability of mimicking all normal positions of the human head. 

**Table 1:** Kodak 9000 CBCT device specifications

Tube voltage	60-90 kilo voltage
Tube current	12-15 milliampere
Frequency	140 kilohertz
Focal spot	0.5 millimetre
Total filtration	2.5 millimetre aluminium
Technology	Digital volumetric tomography
Technology of sensor	CMOS with fiber optic
Volume size	30*50
Voxel size	76*76*76 micrometer

**Imaging**

**Figure 2:**
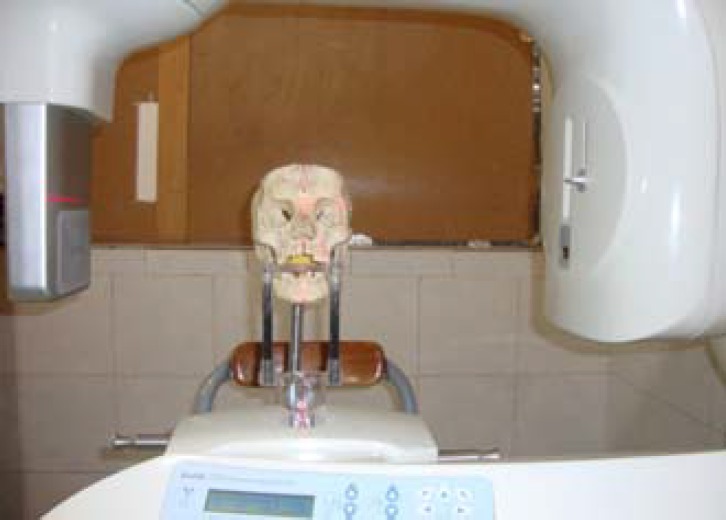
Imaging of the Skull

Kodak 9000 CBCT with the specifications listed in Table 1 was used for imaging. Imaging was performed in the standard position of the skull so the mandibular occlusal plane was parallel to the surface. Midsagittal line which passes anatomic sites such as Nasion, Anterior nasal spine and Pogonion (This line was drawn on the skull), was consistent with the Laser line guide (Figure 2). Imaging performed with 60kvp, 6.3mA and was 10.63s. Then it was repeated at 10 other positions listed as follows:

1. Replacing the skull, 7 mm to the left from the standard position, so that the laser guide line of the device was based on the "f" line.
2. Replacing the skull 7 mm to the right from the standard position, so that the laser guide line of the device based on the "g" line.
3. Replacing the skull 7 mm to the anterior from the standard position so that a reference point located on the bite block was in accord with the line “c".
4. Replacing the skull 7 mm to the posterior from the standard position so that a reference point located on the bite block was in accord with the line "j".
5. Rotation of skull, 10 degrees to the left from the standard position around the vertical axis so that the laser-guideline was in accord with the line "a".
6. Rotation of skull, 10 degrees to the right from the standard position around the vertical axis so that the laser-guideline was in accord with the line "b”.
7. Rotation of skull, 10 degrees up from the standard position around the horizontal axis so that the line "I" was parallel to the ground.
8. Rotation of skull, 10 degrees down from the standard position around the horizontal axis so that the line "h" was parallel to the ground.
9. Rotation d position around the sagittal axis so that the laser of skull, 10 degrees to the left from the standard- guideline was in accord with "d" line.
10. Rotation of skull, 10 degrees to the right from the standard position around the sagittal axis so that the laser-guideline  was in accord with "e" line.

**Figure 3:**
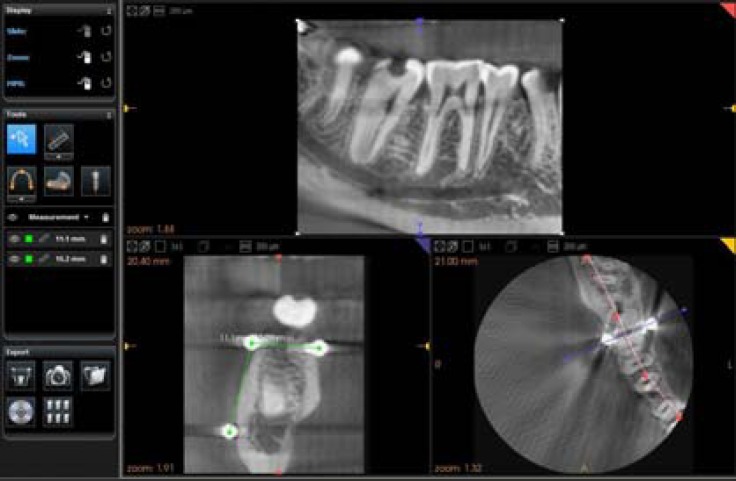
Images obtained from Kodak 9000 software and measurements

Images obtained from the Kodak 9000 software in which axial slices were parallel to the occlusal plane and cross sectional slices were associated with each axial section, were reconstructed (Figure 3). Each section had a thickness of 200 micrometers.

**Measurement**

Two linear measurements were performed between the points determined by the radio opaque markers on the skull. One of them was the transverse distance between markers 1 and 2; the other was the vertical distance be-

tween markers 1 and 3.

The two measurements were performed with Kodak 9000 software. Since the markers were spherical, they were emerging as circles in the sectional images. Therefore the distances between the centers of the circles were measured. These measurements were repeated with pre-planned changes in position (10 aforementioned positions) of the skull (Figure 3).

Finally all the measurements carried out in 10 positions were compared with the measurements in the ​​standard position.

## Results

In the standard position, the transverse distance approximately 11mm and a vertical distance roughly 15mm was measured, The values which are more profound in clinical assessments. Then these measurements were repeated by pre-planned changes in the posture of the skull (10 positions). Values obtained from both the transverse and vertical distances measured in each position were similar to those in the standard situation.  

## Discussion

As the results demonstrated, the values ​​gained from transverse and vertical distances in each position are similar to those in standard position. Our aim of this study was to investigate the effect of all possible pre-planned positions of the dry skull on the accuracy of linear measurements based on the images obtained from CBCT. The basic requisites for employing sectional images, in pre-surgical treatment planning of Implant therapy, are the absence of the spatial distortion to yield dimensional accurateness of the images and the reproducibility of linear measurements. An important issue in evaluating reformatted CT scan images is the likelihood of spatial distortions owing to the anisotropic nature of the voxels (rectangular cuboid) creating the images. CBCT images are created by isotropic voxels. Any reformatting would not cause any distortion and the position of the subject in the scanning device should not influence the accurateness of linear measurements, recorded from the reformatted images. The spatial distortions of the scanning cylinder are small as the voxels that are composing the image are isotropic. From a clinical point of view, this means that the positioning of the patient is not crucial in CBCT given that all structures of interest are included in the scanned volume [17–18]. Many studies enrolled to demonstrate the accuracy of linear measurements in CBCT. Suomalainen et al. [14] demonstrated that the error of linear measurements is even smaller with CBCT (3D Accuitomo) than with (Multiple Slice Computed Tomography) MSCT in the preoperative planning for dental implant treatments. The studies of Kobayashi et al., Loubele et al., Veyre-Goulet et al., and Kamburoğlu et al. showed high accuracy for linear measurements in implant treatment measurements. Kobayashi et al., evaluated the accuracy of measurement of distance using CBCT (3D Accuitomo) and (MSCT). The vertical distance from an allusion point to the alveolar ridge was deliberated in five cadaver mandibles. A considerably smaller quantity error was detected in their study for CBCT than for MSCT. They construed that distance can be measured precisely using CBCT and it is even more accurate than spiral CT [14-15].

It is imperative to point out that patient imaging is somehow dissimilar to the cadaver studies. To the best of our knowledge, only a few studies [9-11], similar to ours, performed to depict the effects of pre-planned changes in the position of human dry skull with CBCT device. All their results suggested that the changes in the position of the skull in the device have not caused any significant effect on the performed measurements. Our study also aimed to examine a wider range of changes in the position of human dry skull on the accuracy of linear measurements compared to the previous studies. Therefore in this study, all minor possible clinical changes in the position of the skull were evaluated. The results of aforementioned studies are consistent with our findings however our survey was conducted more comprehensively. Based on our knowledge, no more studies in this field performed so that we systematically examined the accuracy of measurements on the obtained images with more changes in the position of the skull. Moreover, a concern during imaging with CBCT, that is a doubt to the accuracy of measurements while changes in standard patient's position defined for the device occur, became cleared out. According to our findings it can be confidently expressed that the  images obtained from CBCT which were induced by minor changes in the position of the skull have sufficient accuracy in linear measurements compared to the standard state.

## Conclusion

Since the accuracy of linear measurements on the images obtained from the CBCT device with minor changes in position of the skull compared to standard position is maintained, re-taking of the images in patients with slight changes in the position of the head during imaging is not required. Moreover, our big apprehension about reproducing similar preoperative radiographic images during the follow-up period is resolved.
